# Involvement of the thalamic reticular nucleus in prepulse inhibition of acoustic startle

**DOI:** 10.1038/s41398-021-01363-1

**Published:** 2021-04-24

**Authors:** Qiang-long You, Zhou-cai Luo, Zheng-yi Luo, Ying Kong, Ze-lin Li, Jian-ming Yang, Xiao-wen Li, Tian-ming Gao

**Affiliations:** State Key Laboratory of Organ Failure Research, Key Laboratory of Mental Health of the Ministry of Education, Guangdong-Hong Kong-Macao Greater Bay Area Center for Brain Science and Brain-Inspired Intelligence, Guangdong Province Key Laboratory of Psychiatric Disorders, Department of Neurobiology, School of Basic Medical Sciences, Southern Medical University, Guangzhou, 510515 China

**Keywords:** Neuroscience, Physiology

## Abstract

Thalamic reticular nucleus (TRN) is a group of inhibitory neurons surrounding the thalamus. Due to its important role in sensory information processing, TRN is considered as the target nucleus for the pathophysiological investigation of schizophrenia and autism spectrum disorder (ASD). Prepulse inhibition (PPI) of acoustic startle response, a phenomenon that strong stimulus-induced startle reflex is reduced by a weaker prestimulus, is always found impaired in schizophrenia and ASD. But the role of TRN in PPI modulation remains unknown. Here, we report that parvalbumin-expressing (PV+) neurons in TRN are activated by sound stimulation of PPI paradigm. Chemogenetic inhibition of PV+ neurons in TRN impairs PPI performance. Further investigations on the mechanism suggest a model of burst-rebound burst firing in TRN-auditory thalamus (medial geniculate nucleus, MG) circuitry. The burst firing is mediated by T-type calcium channel in TRN, and rebound burst firing needs the participation of GABAB receptor in MG. Overall, these findings support the involvement of TRN in PPI modulation.

## Introduction

Thalamic reticular nucleus (TRN) is a shell-like structure that is mainly composed of GABAergic neurons. TRN lies between cortex and thalamus, and receives inputs from both cortex and thalamus but sends outputs only to the thalamus^1,2^. Due to its specific position and synaptic connections, TRN is thought to play an important role in sensory information processing, such as sensory detection^3^, sensory selection^4,5^, and sensory gating^6,7^. Aberrant sensory processing is common in some major psychiatry disorders, so TRN is considered as the ideal target for the investigation of the pathophysiology of schizophrenia^8–10^ and autism spectrum disorder (ASD)^11–13^.

Prepulse inhibition (PPI) of the acoustic startle response is a cross-species sensorimotor phenomenon, which refers to the ability of a weak prestimulus to transiently inhibit the response to a closely following strong sensory stimulus^14^. PPI deficit has been known as one of the typical phenotypes of schizophrenia^15,16^ and ASD^17,18^. Although many studies on the mechanism of PPI have been conducted, the relationship between TRN and PPI remains unknown.

Previous studies on the neurobiology of PPI have shown that acoustic startle reflex circuit includes the ventral cochlear nucleus, nuclei of lateral lemniscus, nucleus reticularis pontis caudalis, spinal interneuron, and lower motor neuron^19^. However, recent studies have found that higher brain regions are also involved in the modulation of PPI of acoustic startle reflex, such as auditory cortex and prefrontal cortex^20–22^. This type of top-down modulation of PPI requires a pathway of auditory information transmission, including auditory thalamus, known as medial geniculate nucleus (MG), and auditory projecting TRN (audTRN). The involvement of MG in PPI has already been reported^23^, but there is no study on the involvement of audTRN in PPI. Besides, researchers also found that activation of the inhibitory TRN amplifies the sound response in MG and auditory cortex using in vivo electrophysiological technology^24^. These leave us a very interesting issue of what part of TRN plays in the modulation of PPI.

In the present study, we show that increased PV+ neuronal activity in the audTRN is tightly coupled with sound stimulus of PPI, and inhibition of PV+ neurons in the audTRN impairs PPI performance. Moreover, we introduce a burst-rebound burst firing model between audTRN and MG neurons which regulates PPI performance. These results indicate the essential role of the audTRN–MG circuit for regulating PPI of acoustic startle response.

## Materials and methods

### Animals

C57BL/6J mice (aged 8–12 weeks) were obtained from the Southern Medical University Animal Center (Guangzhou, China). PV-Cre and Ai14 mice were obtained from the Jackson Laboratory. Four to five mice were housed in one plastic cage (30 × 17 × 12 cm) at 24 ± 1 °C. The mice were maintained under standard laboratory conditions (12-h light/dark cycle, lights on from 8:00 a.m. to 8:00 p.m.) with access to food and water ad libitum. Only male mice were used in the study.

### Ethics approval

Animal experiments were conducted following the Regulations for the Administration of Affairs Concerning Experimental Animals (China) and were approved by the Southern Medical University Animal Ethics committee.

### Virus

Adeno-associated viruses (AAVs) used in this study were purchased from BrainVTA, Wuhan, China and included AAV_2/9_-Efla-DIO-GCaMP6f-P2A-NLS-WPRE-pA (titer, 2.86 × 10^12^ v.g./mL), AAV_2/9_-Efla-DIO-hM4Di-P2A-NLS-mCherry-WPRE-pA (titer, 2.50 × 10^12^ v.g./mL), and AAV_2/9_-Efla-DIO-P2A-NLS-mCherry-WPRE-pA (titer, 2.58 × 10^12^ v.g./mL).

### Surgery

Mice were anesthetized with sodium pentobarbital (1% wt/vol) via intraperitoneal injection. Viruses were injected into brain nuclei using stereotaxic equipment (Ruiwode Life Science). All coordinates for the injection sites are listed as measurements from the bregma. AudTRN:1.85 mm posterior, 2.45 mm lateral, 3.35 mm ventral; MG: 2.8 mm posterior, 2.0 mm lateral, and 3.2 mm ventral (for details, see Supplementary Methods).

### Animal behavioral paradigm

#### Open field test (OFT)

The OFT was performed in 40 × 40 cm chambers. The mouse was gently placed in the center of the square for a 5-min recording period. The time spent in the center zone and the distances traveled were automatically calculated using the DigBehv animal behavior analysis program.

#### PPI of startle reflex

As previously described^25^, PPI was measured using the SR-Lab System (San Diego Instruments, San Diego, CA, USA). During all testing, the background was set at 65-dB white noise. The PPI test consisted of a 5 min acclimation period, followed by seven trial types presented six times each in pseudorandom order. Each trial consisted of a 20 ms prepulse, an 100 ms interval, and a 40 ms startle stimulus. Prepulse intensity was set at background, 74, 78, 82, 86, or 90 dB, and the startle stimulus in each case was 120 dB. One trial type consisted solely of background noise to control for the general movement of the subject. Trials were presented with a variable intertrial interval of 15–20 s. For each subject, the entire PPI test session lasted approximately 18 min. At the onset of the startle stimulus, maximum velocity (*V*_max_) in arbitrary units of the startle platform was recorded every 1 ms for 65 ms. The *V*_max_ was averaged across trial type, with PPI calculated as the difference between *V*_max_ after prepulse trials (*V*_max-PP_) and *V*_max_ after startle stimulus alone trials (*V*_max-startle_), and was expressed as a percentage of *V*_max-startle_: PPI = ((*V*_max-startle_ − *V*_max-PP_)/*V*_max-startle_)×100%.

### Fiber photometry

Mice were allowed to recover from surgery for 14 days before the behavioral experiments. The fiber photometry system (ThinkerTech Nanjing Bioscience) was used in our study (for details, see Supplementary Methods). During the behavior experiment, the GCaMP6f fluorescence intensity from startle white noise (120 dB) and prepulse white noise (90 dB) was recorded. The signal during background white noise (65 dB) was set as the baseline.

For all behavioral tests, mice were separated into each group (*N* ≥ 8 mice) randomly. Analyses were performed blindly. All experiments were replicated more than three times in the laboratory.

### In vitro electrophysiological recording

After slice preparation (refer to Supplementary Methods for details), slices were placed in the recording chamber that was superfused (3 mL/min) with ACSF at 30–32 °C. Whole-cell patch-clamp recordings of TRN and MG neurons were obtained under an infrared (IR)-differential interference contrast (DIC) microscope (Nikon). To record action potentials, pipettes (input resistance: 3–7 MΩ) were filled with an intracellular solution containing (in mM) 105 K-gluconate, 30 KCl, 10 HEPES, 10 phosphocreatine, 4 ATP-Mg, 0.3 GTP-Na, and 0.3 EGTA (pH 7.35, 290 mOsm). Depolarizing currents (60 pA, 500 ms) were injected into PV-positive neurons in audTRN of PV-Cre: Ai14 mice in the current-clamp configuration, and a recording was conducted at holding potentials of −70 mV. To isolate T-type calcium channel-dependent low threshold spike (LTS), 1 µM tetrodotoxin (TTX) was added to the ACSF. To block this LTS, T-type calcium channel antagonist NiCl2 (1 mM, Sigma Aldrich) was added to the ACSF. When the inhibitory synaptic response was recorded, a stimulating electrode was placed in the audTRN fiber path approximately 0.2 mm away from the recorded cell bodies in the MG. The electrical stimulation frequency was 300 Hz (0.2 and 50 ms), and a recording was conducted at holding potentials of −60 mV. To isolate the synaptic IPSPs, 20 μM CNQX and 100 μM D,L-APV were added to the ACSF. After the burst firing was induced, 20 µM GABAA receptor antagonist bicuculline methiodide (BMI) and 0.5 µM GABAB antagonist CGP55845 (ref. ^26^) were added to the ACSF. Data were recorded with a multiClamp 700B (Molecular Devices) software, digitized at 5 kHz, and filtered at 1 kHz. Data were collected when the series resistance fluctuated within 20% of the initial values and analyzed using pClamp10.7 software (Molecular Devices).

### Immunostaining

For histological procedures, please refer to the Supplementary Materials.

### Statistical analyses

Statistical comparisons were conducted in GraphPad Prism 8. The homoscedasticity and normality of the distributions of data were determined using GraphPad Prism 6 before assigning specific statistical tests. Data were analyzed using Student’s *t*-test or Mann–Whitney two-tailed *t*-test or two-way analysis of variance (ANOVA). Data are expressed as the means ± standard errors of the means (SEM), unless otherwise indicated, and a difference was considered statistically significant when *P* < 0.05. The sample size (*N* = number of neurons or mice) and statistical test results, such as *p, t*, and *df* are reported in results for all measurements.

## Results

### PV+ neurons in auditory TRN are activated during PPI process

**Fig. 1:**
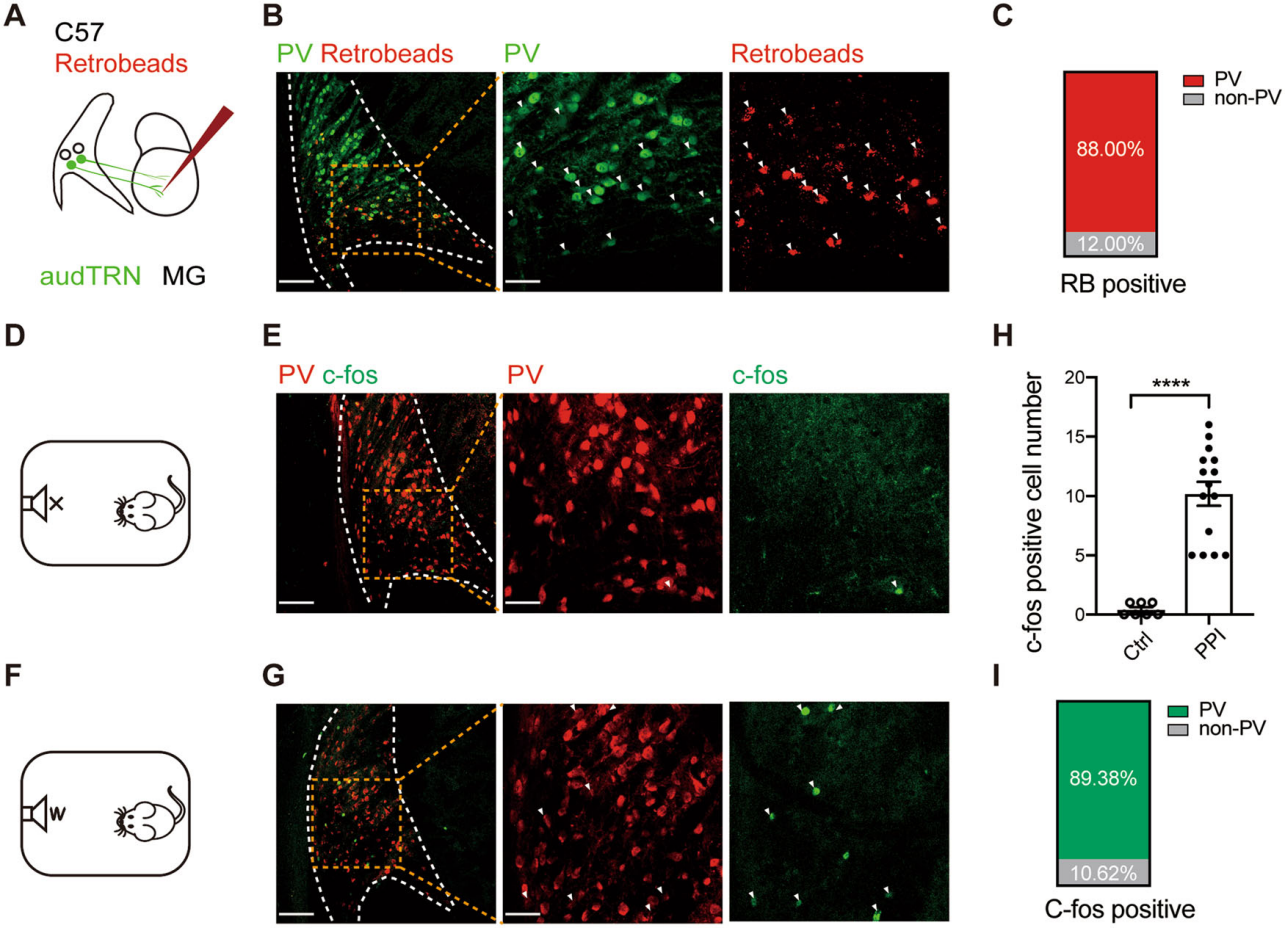
PPI paradigm increases c-Fos expression of PV+ neurons in audTRN. **A** Strategy of retrograde labeling of audTRN neurons projecting to MG. Retrobeads (RBs) were injected into MG of C57 mice. **B** Left, example of confocal image showing overlap of retrobeads-labeled neurons(red) and PV-positive neurons (green). White dotted lines mark the border of the TRN. Middle and right, enlarged images from the yellow box region in the left image. Arrows: dual-positive neurons. Scale bars: left to right, 100 µm, 40 µm. **C** Quantification shows that approximately 88.0% of RBs labeled cells are PV+ (*N* = 7 slices of 3 mice). **D**, **F** Schematic of the behavioral test before c-Fos staining experiment. C57 mice were put into the startle reflex chamber without sound stimulation (**D**, control group), or with prepulse inhibition (PPI) paradigm (**F**, PPI group). **E**, **G** Left, example of confocal image showing overlap of c-Fos-positive neurons and PV-positive neurons of control group (**E**), or PPI group (**G**). White dotted lines mark the border of the TRN. Middle and right, enlarged images from the yellow box region in the left image. Arrows: dual-positive neurons. Scale bars: left to right, 100 µm, 40 µm. **H** PPI paradigm increased c-Fos-positive neuron number compared with the control group (Mann–Whitney two-tailed *t*-test, *P* < 0.0001, *N*_control_ = 7 slices of 3 mice, *N*_PPI_ = 15 slices of 6 mice). **I** Quantification shows that approximately 89.38% of c-Fos-positive cells are PV+ (*N* = 15 slices of 6 mice, PPI group). ****P < 0.0001.

In consideration of the fact that TRN has several subsections related to different types of sensory information processing^1^, we first focused on the auditory pathway for its wide usage in studies on PPI. To identify the position and cell type of auditory thalamus projecting TRN (audTRN), we injected retrobeads (RBs) into the MG of C57 mice (Fig. 1A and Supplementary Fig. S1). After the position of audTRN was confirmed (Fig. 1B), we tested whether parvalbumin-expressing (PV+) neurons are predominated as previously described^27^ by calculating the ratio of the co-label between RBs and PV+ neurons in audTRN. The result showed that about 88.00 ± 2.23% MG-projecting neurons in audTRN were PV positive (Fig. 1B, C), which indicated that PV+ neurons account for the majority of the auditory pathway of TRN.

Next, we determined whether PV+ neurons are activated during the sound stimuli of PPI paradigm. C57 mice were randomly divided into two groups. Mice were put into the startle reflex chamber without or with sound stimuli of PPI (Fig. 1D, F). After c-Fos staining, we found that PPI paradigm significantly increased c-Fos expression compared to the control group (Fig. 1H; number of c-Fos-positive neurons, control: 0.43 ± 0.20, PPI: 10.20 ± 1.01; *P* < 0.0001). The result revealed that audTRN was activated during sound stimuli of PPI. Among the c-Fos-positive neurons of the PPI group, approximately 89.38 ± 4.40% neurons were PV+ (Fig. 1I).

**Fig. 2:**
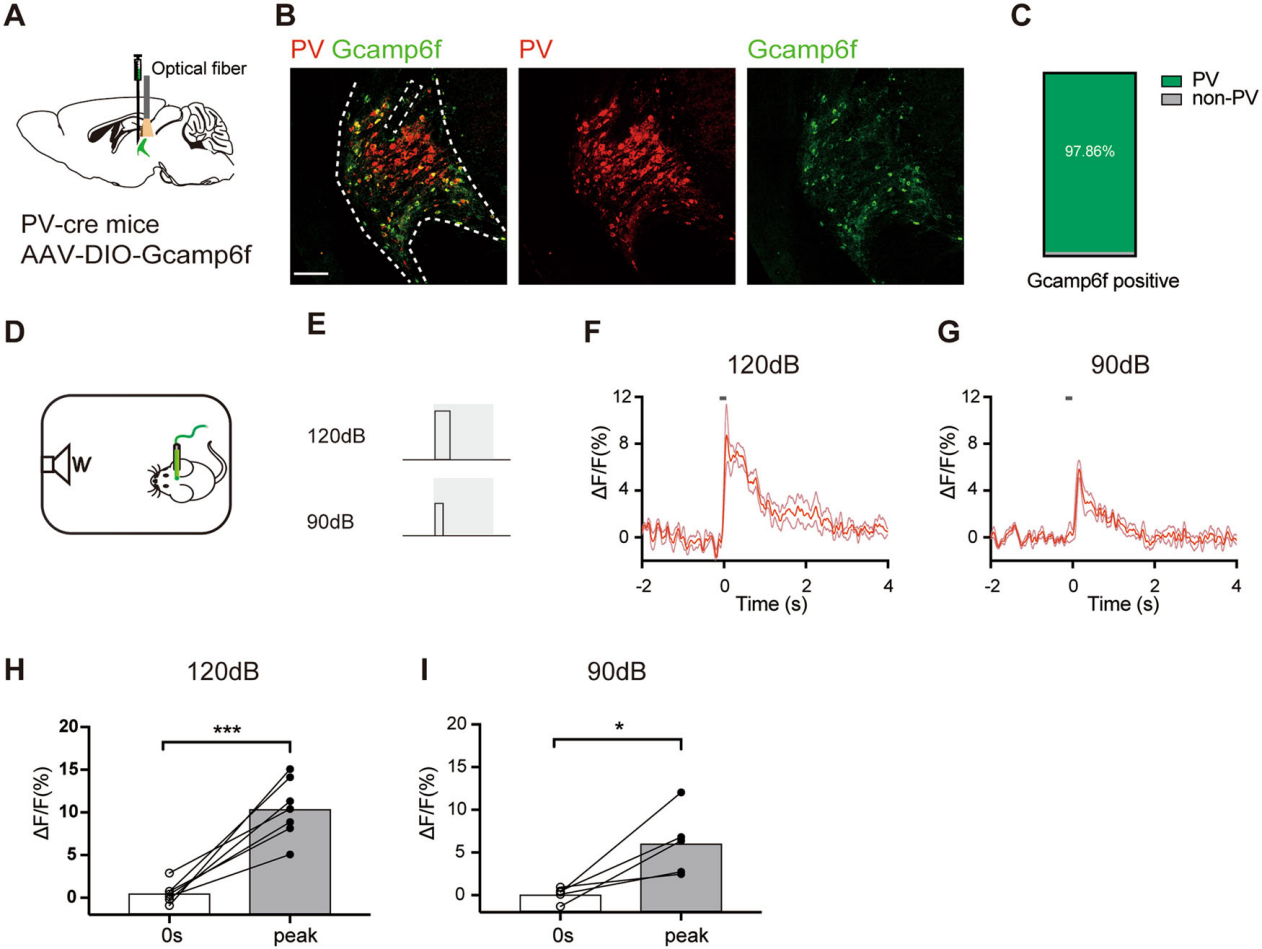
Sound stimuli of PPI paradigm increases calcium activity of PV+ neurons in audTRN. **A** Strategy of calcium activity recording of PV+ neurons in audTRN. A Cre-dependent AAV for expression of Gcamp6f was bilaterally injected into the audTRN in PV-cre mice. **B** Left, example of confocal image showing overlap of Camp6f+ neurons (green, right) and PV+ neurons (red, middle). White dotted lines mark the border of the audTRN. Scale bar: 100 µm. **C** Quantification shows that approximately 97.86% of Gcamp6f cells are PV+ (*N* = 3 mice). **D**, **E** Schematic of calcium activity recording experiment. Mice were given sound stimuli by a speaker in a sound-proof chamber. The time duration of the sound stimulus was 40 ms (120 dB) and 20 ms (90 dB). **F**, **G** Examples of calcium responses in the audTRN when mice were given sound stimuli (**F**, 120 dB; **G**, 90 dB). Thick lines indicate the average, and thin lines indicate the s.e.m. A short line (black) in each image indicates the sound stimulus period. **H**, **I** Sound stimuli of PPI paradigm Increased calcium activity in audTRN (**H**, 120 dB, paired two-tailed *t*-test, *t*_6_ = 6.723, *P* = 0.0005, *N* = 7 mice) (**I** 90 dB, paired two-tailed *t*-test, *t*_4_ = 3.273, *P* = 0.0307, *N* = 5 mice), *P < 0.05, ***P < 0.001.

Given that c-Fos staining was carried out in slices in vitro, we further determined whether PV+ neuronal activation appears in audTRN in vivo during sound stimuli of PPI using fiber photometry. A Cre-dependent AAV for expression of the Ca^2+^ indicator Gcamp6f, AAV-DIO-GCaMP6f, was injected into audTRN in PV-Cre mice (Fig. 2A, B). Among the infected neurons, about 97.86 ± 0.93% neurons expressed PV (Fig. 2C). Different sound stimuli of PPI, 90 and 120 dB, were generated by a white noise generator and were given by a speaker in a behavioral chamber where ca2+ fluorescence (∆*F*/*F*(%)) was monitored by fiber photometry system (Fig. 2D). We found that GCaMP6f fluorescence in PV+ neurons in the audTRN increased when mice were presented with startle 120 dB sound stimulation (Fig. 2F, H. ∆*F*/*F*(%), 0 s: 0.55 ± 0.45, peak amplitude: 10.42 ± 1.31; *P* = 0.0005), and 90 dB sound stimulation (Fig. 2G, I; ∆*F*/*F*(%), 0 s: 0.11 ± 0.38, peak amplitude: 6.08 ± 1.74; *P* = 0.0307). Taken together, these results indicate that the activity of PV+ neurons in audTRN is tightly coupled with the sound stimuli of PPI.

### Chemogenetic inhibition of audTRN PV neurons impairs PPI performance

**Fig. 3:**
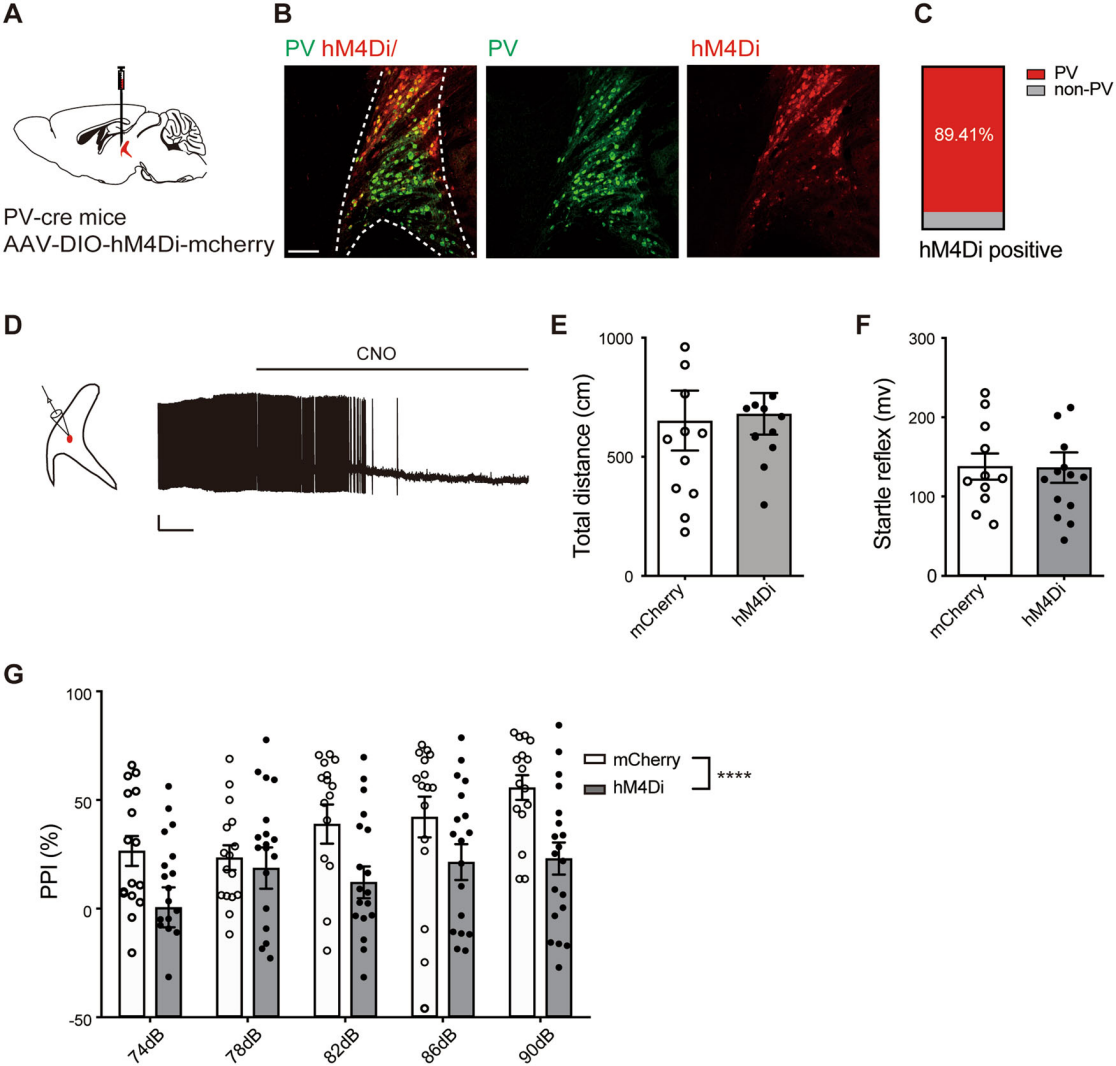
Chemogenetic inhibition of PV+ neurons in audTRN impairs PPI performance. **A** Strategy of chemogenetic inhibition of PV+ neurons in audTRN. A Cre-dependent AAV for expression of hM4Di was bilaterally injected into the audTRN in PV-cre mice. **B** Left, example of confocal image showing overlap of hM4Di+ neurons (red, right) and PV+ neurons (green, middle). White dotted lines mark the border of the audTRN. Scale bar: 100 µm. **C** Quantification shows that approximately 89.41% of hM4Di cells are PV+ (*N* = 9 slices of 3 mice). **D** Left, schematic of experiment design. Right, a representative trace recorded in current-clamp mode from an audTRN PV neuron that expressed hMD4i. Scale bars: 1 min, 20 mV. Application of CNO (5 µM) abolished neuronal firing. **E**, **F** Application of CNO (3 mg/kg, i.p.) to mice that expressed hMD4i in audTRN PV neurons had no effect on total distance in OFT (Student’s *t*-test, *t*_22_ = 0.1871, *P* = 0.8533, *N* = 12 mice per group) or startle reflex in PPI (Student’s *t*-test, *t*_23_ = 0.0559, *P* = 0.9559, *N*_mCherry_ = 11 mice, *N*_hM4Di_ = 14 mice). **G** Application of CNO (3 mg/kg) to mice that expressed hMD4i in audTRN PV neurons impaired PPI performance (two-way ANOVA, *F*_(1,165)_ = 18.90, *P* < 0.0001, *N*_mCherry_ = 16 mice, *N*_hM4Di_ = 19 mice). ****P < 0.0001.

To determine the role of PV+ neuronal activity in audTRN in mediating PPI performance, we examined the effect of chemogenetic inhibition of PV+ neurons. An AAV virus for expression of the engineered inhibitory G protein-coupled receptor (hM4Di), AAV-DIO-hM4Di-mCherry, or a control virus, AAV-DIO-mCherry, was injected into the audTRN in PV-Cre mice (Fig. 3A). Among the infected neurons, about 89.41 ± 1.08% neurons expressed PV (Fig. 3C). HM4Di suppressed neuronal activity in the presence of its agonist clozapine-N-oxide (CNO). The efficacy of hM4Di-mediated inhibition was tested in brain slices using whole-cell recordings from PV+ neurons expressing hM4Di (Fig. 3D). Infusions of CNO (5 µM) into audTRN slices from these mice resulted in blockage of action potential firing. For behavioral test, after CNO (3 mg/kg, i.p.) treatment, the hM4Di group showed no effect on the total distance of OFT (Fig. 3E and Supplementary Fig. S2), or startle reflex of PPI (Fig. 3F) compared to the mCherry group, which indicated that inhibition of PV+ neuron in audTRN does not affect the locomotor activity or startle system. In contrast, CNO treatment decreased the PPI of the hM4Di group significantly compared to the mCherry group (Fig. 3G, *P* < 0.0001). Together, these data indicated that the PV+ neurons in audTRN are critical for PPI of acoustic startle.

### Inhibition of T-type calcium channel causes blockage of burst firing and PPI performance impairment

Previous studies have found that neurons in the sensory system exhibit two different firing modes, tonic and burst firing^28,29^. Tonic firing affords better linearity, whereas burst firing supports better signal detection^30,31^. Sound stimulation can induce burst firing in TRN^3,6^, and the activity of PV+ neurons in audTRN is tightly coupled with the sound stimuli of PPI (Fig. 2), we then hypothesized that burst firing of PV+ neurons in audTRN may be involved in the process of PPI modulation.

**Fig. 4:**
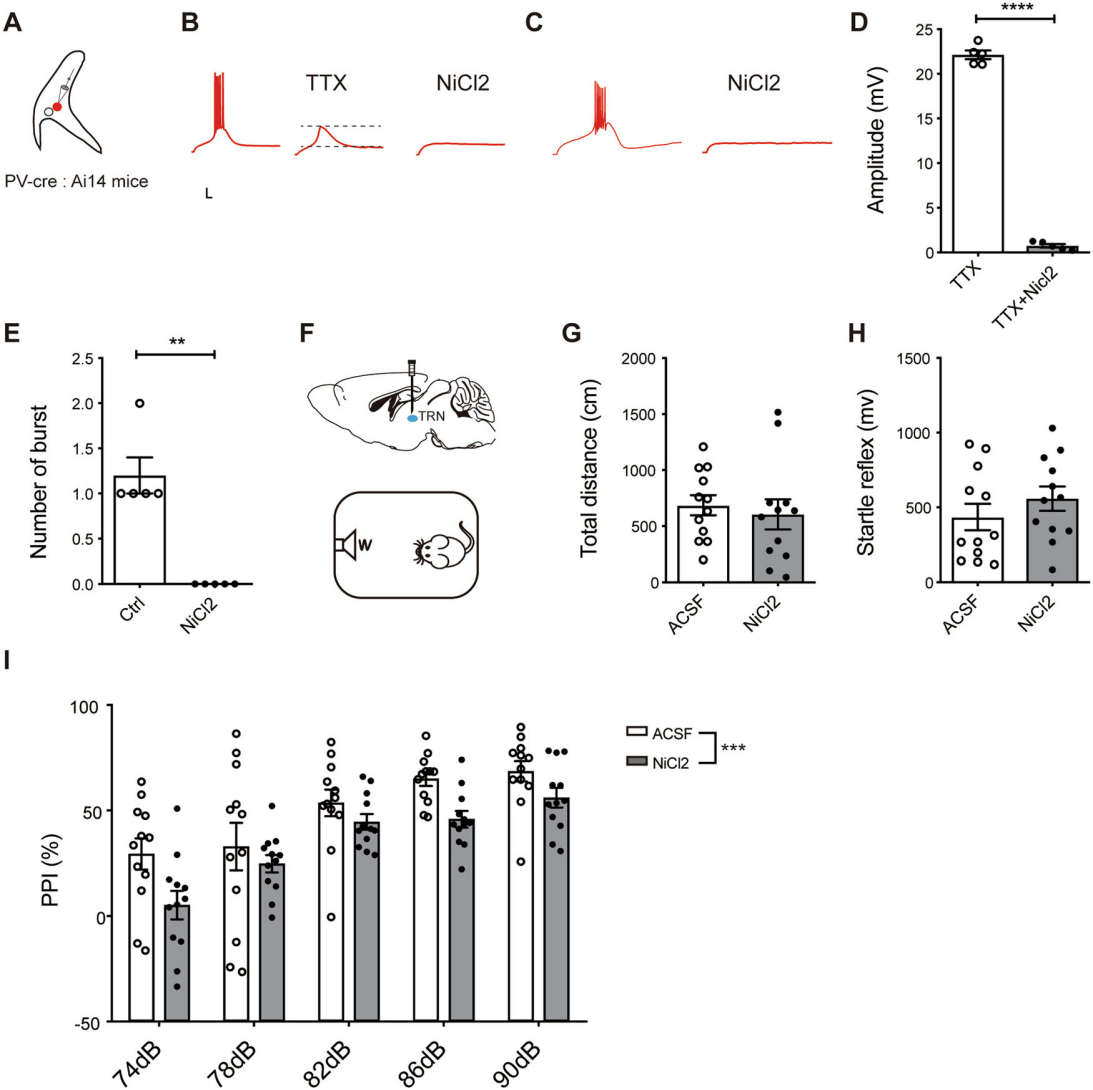
NiCl2 causes blockage of burst firing of PV+ neurons in audTRN and PPI performance impairment. **A** Electrophysiological recording in audTRN slice of PV-cre: Ai14 mice. **B** Left, example of burst firing induced by 60 pA inward current injection. Middle, action potential was blocked by 1 µM TTX. The spike between two dotted lines was T-type calcium channel spike, which was suppressed by 1 mM NiCl2 (right). Scale bars: 10 ms, 10 mV. **C** Burst firing was blocked by 1 mM NiCl2. **D** Quantification of the calcium spike amplitude from the TTX and TTX + NiCl2 group. NiCl2 blocked the calcium spike (paired two-tailed *t*-test, *t*_4_ = 31.390, *P* < 0.0001, *N* = 5 cells). **E** Quantification of the burst number from the control and NiCl2 group. NiCl2 blocked burst firing completely (paired two-tailed *t*-test, *t*_4_ = 6.000, *P* = 0.0039, *N* = 5 cells). **F** Schematic of pharmacological experiment. Cannulas were unilaterally implanted into audTRN of C57 mice. After 7 days recovery, mice were given 6 mM NiCl2 or ACSF 30 min before OFT and PPI paradigm. **G**, **H** Infusion of 6 mM NiCl2 into audTRN of mice had no effect on total distance in OFT (Student’s *t*-test, *t*_22_ = 0.5074, *P* = 0.6169, *N* = 12 mice per group) or startle reflex in PPI (Student’s *t*-test, *t*_22_ = 1.027, *P* = 0.3158, *N* = 12 mice per group). **I** Infusion of 6 mM NiCl2 into audTRN of mice impaired PPI performance (two-way ANOVA, *F*_(1,110)_ = 14.480, *P* = 0.0002, *N* = 12 mice per group). **P < 0.01, ***P < 0.001, ****P < 0.0001.

To testify this idea, we first confirmed that PV+ neurons in audTRN can fire in burst ways using whole-cell recordings from PV+ neurons of PV-Cre: Ai14 mice by inward current injection (Fig. 4A, B). It has been shown that burst firing was triggered by T-type calcium channel-mediated depolarization, known as the LTS^29,32^. LTS could be isolated by the addition of voltage-gated sodium channel blocker TTX (Fig. 4B). To prove the leading role of T-type calcium channel in burst firing, its antagonist NiCl2 was added into the ACSF^33^. One millimolar NiCl2 could block LTS (Fig. 4B, D, amplitude of LTS, TTX: 22.43 + 0.19 mV, TTX + NiCl2: 0.75 + 0.20 mV; *P* < 0.0001) as well as burst firing (Fig. 4C, E, number of burst, control: 1.2 + 0.20, NiCl2: 0; *P* = 0.0039) of PV+ neurons in audTRN. The results showed that NiCl2 attenuated the burst firing of PV+ neurons efficiently.

To test the involvement of burst firing in the PPI modulation, 6 mM NiCl2 (ref. ^33^) or vehicle was injected into audTRN of C57 mice by the micro-injection system (Fig. 4F and Supplementary Fig. S1). NiCl2 treatment showed no effect on the total distance of OFT (Fig. 4G and Supplementary Fig. S2), or startle reflex of PPI (Fig. 4H) compared to vehicle group, indicating that inhibition of burst firing in audTRN does not affect the locomotor activity or startle system. In contrast, NiCl2 treatment decreased PPI significantly compared to the vehicle group (Fig. 4I, *P* = 0.0002). These results inferred that burst firing of audTRN neurons plays an important role in the regulation of PPI of startle reflex.

### Inhibition of GABAB receptor prevents rebound bursting in the MG and impairs PPI of acoustic startle

How does the burst firing in audTRN regulate PPI performance of startle reflex? TRN is an inhibitory nucleus which sends inhibitory outputs to thalamic relay cells. Burst firing of interneurons in TRN can provide a short but strong hyperpolarization for relay cells. Previous studies have found that thalamic relay cells’ bursts potently activate cortical circuits^31,34^ served as a “wake-up call” from thalamus^30^. This suggests that burst firing of thalamic relay cells is responsible for the detection of sensory information. So we wondered whether bursting in audTRN neurons regulates burst firing in thalamic relay neurons.

**Fig. 5:**
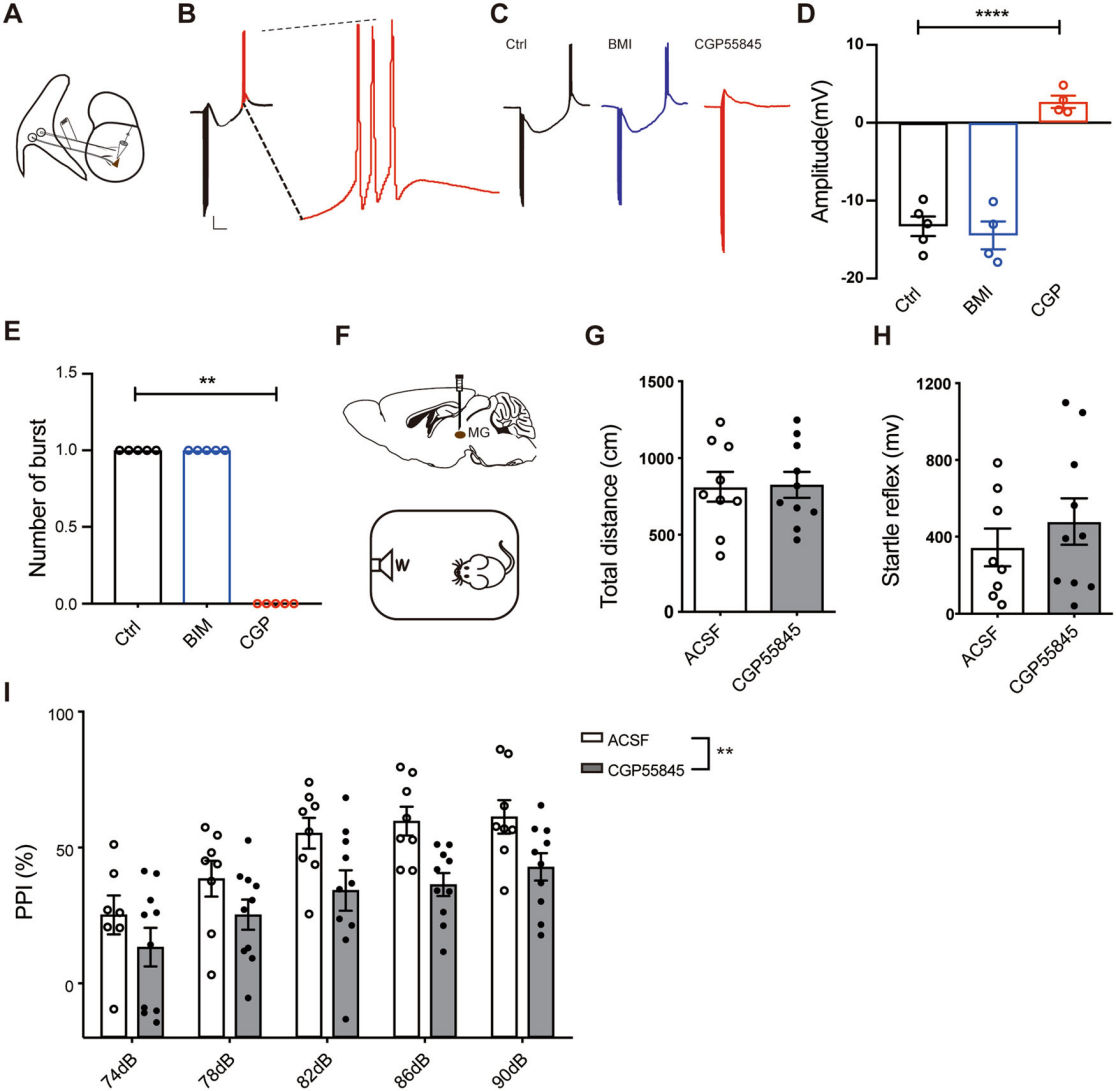
Inhibition of GABAB receptor prevents rebound bursting in the MG and impairs PPI performance. **A** Electrophysiological recording in MG slice of PV-cre: Ai14 mice. A stimulating electrode was placed in the audTRN fiber path. The electrical stimulation frequency was 300HZ. **B** Left, example of burst firing induced by electrical stimuli of the inhibitory audTRN–MG pathway. 100 μM APV and 20 μM CNQX were used to block excitatory input. The amplitude of hyperpolarization was calculated between two dotted horizontal lines. Right, enlarged trace from the dotted line area in the left trace. Scale bars: 100 ms, 10 mV. **C** Example trace of rebound burst firing without drug treatment (left, group_control_), with GABAA receptor antagonist 20 μM BMI treatment (middle, blue, group_BMI_), or with GABAB antagonist 0.5 μM CGP55845 treatment (right, red, group_CGP_). **D**, **E** Hyperpolarization and rebound burst firing were blocked by GABAB receptor antagonist but not by GABAA antagonist (**D** amplitude of hyperpolarization, group_control_ versus group_CGP_, Student’s *t*-test, *t*_7_ = 10.110, *P* < 0.0001; **E** number of burst firing, group_control_ versus group_CGP_, Mann–Whitney two-tailed *t*-test, *P* = 0.0079, *N* = 5 cells). **F** Schematic of pharmacology experiment. Cannulas were unilaterally implanted into MG of C57 mice. After 7 days recovery, mice were given 0.1 mM CGP55845 or ACSF 30 min before OFT and PPI paradigm. **G**, **H** Infusion of 0.1 mM CGP55845 to MG of mice had no effect on total distance in OFT (Student’s *t*-test, *t*_17_ = 0.103, *P* = 0.9190, *N*_ACSF_ = 9 mice, *N*_CGP_ = 10 mice) or startle reflex in PPI (Student’s *t*-test, *t*_16_ = 0.834, *P* = 0.4163, *N*_ACSF_ = 8 mice, *N*_CGP_ = 10 mice). **I** Infusion of 0.1 mM CGP55845 to MG of mice impaired PPI performance (two-way ANOVA, *F*_(1,80)_ = 8.733, *P* = 0.0041, *N*_ACSF_ = 8 mice, *N*_CGP_ = 10 mice). **P < 0.01,****P < 0.0001.

First, an electrical stimulus was given by a stimulating electrode placed in the audTRN fiber path while whole-cell recording was conducted in relay neurons in MG. To mimic the burst firing in audTRN, the electrical stimulation parameters were set up as follows: 300 Hz, 50 ms duration, 0.2 ms pulse width^28,35,36^. CNQX, and APV were added to the ACSF to block the effect of excitatory synaptic inputs (Fig. 5A). We found that after a strong hyperpolarization from inhibitory synaptic inputs of TRN, MG relay neurons exhibited a rebound burst firing^28^ afterward (Fig. 5B). Furthermore, this kind of hyperpolarization and rebound burst could be blocked by GABAB receptor antagonist CGP55845, rather than GABAA receptor antagonist bicuculline methiodide (BMI) (Fig. 5C–E). These data suggest that GABAB receptor-mediated hyperpolarization plays an important role in triggering rebound burst firing in MG. Next, we used CGP55845 to block the rebound burst in MG in the behavioral tests. In all, 0.1 mM CGP55845 or vehicle was injected into the MG of C57 mice by the micro-injection system (Fig. 5F and Supplementary Fig. S1). CGP55845 treatment showed no effect on the total distance of OFT (Fig. 5G and Supplementary Fig. S2), or startle reflex of PPI (Fig. 5H) compared to the vehicle group, indicating that inhibition of rebound burst firing of neurons in MG does not affect the locomotor activity or startle system. In contrast, CGP55845 treatment decreased PPI significantly compared to the vehicle group (Fig. 5I, *P* < 0.0001). All these data suggested that there may exist a burst-rebound firing pathway from audTRN to MG and this pathway is critical to the regulation of PPI of startle reflex.

## Discussion

Using cell-type-specific fiber photometry, electrophysiology, chemogenetics, and pharmacology, we report three major findings. First, PV+ neurons in the audTRN are activated by sound stimulation. Second, chemogenetic inhibition of PV+ neurons in the audTRN impairs PPI performance. Last, pharmacological inhibition of burst firing in audTRN, or rebound burst firing in MG also impairs PPI performance. Together, these findings revealed an audTRN–MG circuit that controls PPI of acoustic startle.

What do these findings inspire us about the relationship between TRN and psychiatry disorders? Previous studies on TRN and sensory gating were often conducted using anesthetized animals^3,6^. Lack of in vivo experimental evidence makes TRN’s role in PPI of acoustic startle unclear. To overcome this obstacle, we first identified the position of auditory related TRN to obtain a better recording of neuronal activity. Then we found that Gcamp6f fluorescence in PV+ neurons in audTRN was increased during sound stimulation of PPI in awake mice, suggesting the involvement of TRN in PPI. Furthermore, the participation of PV+ neurons in the modulation of PPI performance in our chemogenetic study is consistent with a previous human postmortem study in schizophrenia^10^. They found a marked decrease in the number of PV neurons in TRN of schizophrenia patients. All these findings suggest that PV+ neuron abnormalities are a potential cellular mechanism of schizophrenia. As to the relationship between TRN and ASD, plenty of studies have been done. TRN may be involved with the hyperactivity, attention deficits, and hypersensitivity of ASD^11–13^. An animal model of autism, the PTCHD1 knockout mouse, was reported. During development, Ptchd1 is selectively enriched in TRN. In their study, the PTCHD1 knockout mice showed reduced repetitive bursting of TRN neurons, but no change in PPI performance^12^. In consideration of our findings, different kinds of burst firing modes, like single burst firing or repetitive burst firing, may modulate sensory processing in different ways^2,37^. Undoubtedly, complete blockage of burst firing can impair PPI performance.

Burst firing is a popular firing mode in the sensory system. It has always been shown in thalamus during sleep and epilepsy^38,39^. Recent studies also found the appearance of burst firing in awake animals, such as cat and monkey^34,40–42^. In our study, blockage of the burst firing in TRN using T-type calcium channel antagonist disrupted PPI performance in awake mice. This testifies that burst firing may also be functional during awake states. Along with MG, we proposed a burst-rebound burst model that provides a new way of sensory information transmission. In this model, the rebound burst of auditory thalamus may increase cortical detection^31,34^, which in turn amplifies the auditory signal. This kind of rebound excitation was common in the rhythmic brain activity of asleep animals or anesthetized animals^38,43^, but little is known in awake animals. So further investigation of this model is needed, especially the role of the rebound burst in information processing during wakeful periods. Besides, rebound burst firing in MG relies on the GABAB receptor. This is understandable because the activation of the T-type calcium channel requires a slow time course of the GABAB synaptic response^44,45^. Together, these findings provide some molecular targets for the following studies on this issue.

How does the burst-rebound burst firing model regulate PPI of acoustic startle response? Crick F.^46^ has proposed a “searchlight hypothesis” about how TRN participates in the process of attention a long time ago and considered TRN as the gatekeeper of the thalamus. Recent studies on the mechanism of attention have found that TRN can filter out irrelevant information through its inhibition of the sensory thalamus^4,47,48^. Our studies suggest that instead of inhibiting the auditory signal, TRN amplifies the signal through a burst-rebound burst firing model which may work as follows: prepulse sound signal induces burst firing in audTRN PV+ neurons, and this kind of firing of inhibitory terminals produces strong hyperpolarization on MG neurons through GABAB receptors, which in turns activates T-type calcium channel and then triggers burst firing in these MG neurons. This kind of firing can be detected by higher brain regions, which can raise the attention level of an animal. Higher attention level can in turn inhibits following strong sound stimulation^49^, at last, this inhibition produces the PPI effect. In short, prepulse stimulation activates the top-down regulation of PPI through a burst-rebound burst firing model between audTRN and MG.

There are several limitations to our studies. Limited by the technology, we cannot record the firing activities of TRN, MG, or cortex in awake mice. Monitoring analysis of the firing properties in these regions is unavailable so that we cannot investigate the relationship between firing mode and animal behaviors. We are unable to record the neuronal activity of audTRN and MG in vivo at the same time either. This makes it impossible to prove the existence of a burst-rebound burst firing model between audTRN and MG in awake mice. Besides, in consideration of the fact that stimulation given by the bipolar electrode may unavoidably activate other inhibitory pathways to MG in the electrophysiological recording experiment, more specific methods such as optogentics will be used to verify such circuit in the future study.

In summary, our work identifies audTRN PV+ interneurons as an important cellular modulator in the regulation of PPI of acoustic startle response. The burst firing in the thalamus may be the mechanism underlying this kind of modulation. AudTRN PV+ neurons and its firing mode may, thus, serve as potential targets for the treatment of patients with schizophrenia and ASD.

## Supplementary information

Supplementary materials

## References

[1] Pinault D (2004). The thalamic reticular nucleus: structure, function and concept. Brain Res. Brain Res. Rev..

[2] Clemente-Perez A (2017). Distinct thalamic reticular cell types differentially modulate normal and pathological cortical rhythms. Cell Rep..

[3] Yu XJ, Xu XX, He S, He J (2009). Change detection by thalamic reticular neurons. Nat. Neurosci..

[4] Wimmer RD (2015). Thalamic control of sensory selection in divided attention. Nature.

[5] Ahrens S (2014). ErbB4 regulation of a thalamic reticular nucleus circuit for sensory selection. Nat. Neurosci..

[6] Krause M, Hoffmann WE, Hajos M (2003). Auditory sensory gating in hippocampus and reticular thalamic neurons in anesthetized rats. Biol. Psychiatry.

[7] McAlonan K, Brown VJ, Bowman EM (2000). Thalamic reticular nucleus activation reflects attentional gating during classical conditioning. J. Neurosci..

[8] Ferrarelli F, Tononi G (2011). The thalamic reticular nucleus and schizophrenia. Schizophr. Bull..

[9] Pratt JA, Morris BJ (2015). The thalamic reticular nucleus: a functional hub for thalamocortical network dysfunction in schizophrenia and a target for drug discovery. J. Psychopharmacol..

[10] Steullet P (2018). The thalamic reticular nucleus in schizophrenia and bipolar disorder: role of parvalbumin-expressing neuron networks and oxidative stress. Mol. Psychiatry.

[11] Nakajima M, Schmitt LI, Feng G, Halassa MM (2019). Combinatorial targeting of distributed forebrain networks reverses noise hypersensitivity in a model of autism spectrum disorder. Neuron.

[12] Wells MF, Wimmer RD, Schmitt LI, Feng G, Halassa MM (2016). Thalamic reticular impairment underlies attention deficit in Ptchd1(Y/-) mice. Nature.

[13] Krol A, Wimmer RD, Halassa MM, Feng G (2018). Thalamic reticular dysfunction as a circuit endophenotype in neurodevelopmental disorders. Neuron.

[14] Hoffman HS, Ison JR (1980). Reflex modification in the domain of startle: I. Some empirical findings and their implications for how the nervous system processes sensory input. Psychol. Rev..

[15] Javitt DC, Freedman R (2015). Sensory processing dysfunction in the personal experience and neuronal machinery of schizophrenia. Am. J. Psychiatry.

[16] Braff DL, Geyer MA (1990). Sensorimotor gating and schizophrenia. Hum. Anim. Model. Stud. Arch. Gen. Psychiatry.

[17] Perry W, Minassian A, Lopez B, Maron L, Lincoln A (2007). Sensorimotor gating deficits in adults with autism. Biol. Psychiatry.

[18] Frankland PW (2004). Sensorimotor gating abnormalities in young males with fragile X syndrome and Fmr1-knockout mice. Mol. Psychiatry.

[19] Davis M, Gendelman DS, Tischler MD, Gendelman PM (1982). A primary acoustic startle circuit: lesion and stimulation studies. J. Neurosci..

[20] Du Y, Wu X, Li L (2011). Differentially organized top-down modulation of prepulse inhibition of startle. J. Neurosci..

[21] Tóth A (2017). Neuronal coding of auditory sensorimotor gating in medial prefrontal cortex. Behav. Brain Res..

[22] Tapias-Espinosa C (2019). Schizophrenia-like reduced sensorimotor gating in intact inbred and outbred rats is associated with decreased medial prefrontal cortex activity and volume. Neuropsychopharmacology.

[23] Zhang J, Engel JA, Ericson M, Svensson L (1999). Involvement of the medial geniculate body in prepulse inhibition of acoustic startle. Psychopharmacology (Berl.).

[24] Aizenberg M (2019). Projection from the amygdala to the thalamic reticular nucleus amplifies cortical sound responses. Cell Rep..

[25] Kivimae S (2011). Abnormal behavior in mice mutant for the Disc1 binding partner, Dixdc1. Transl. Psychiatry.

[26] Saunders A (2015). A direct GABAergic output from the basal ganglia to frontal cortex. Nature.

[27] Dong P (2019). A novel cortico-intrathalamic circuit for flight behavior. Nat. Neurosci..

[28] Krahe R, Gabbiani F (2004). Burst firing in sensory systems. Nat. Rev. Neurosci..

[29] Sherman SM (2001). Tonic and burst firing: dual modes of thalamocortical relay. Trends Neurosci..

[30] Sherman SM (2001). A wake-up call from the thalamus. Nat. Neurosci..

[31] Swadlow HA, Gusev AG (2001). The impact of ‘bursting’ thalamic impulses at a neocortical synapse. Nat. Neurosci..

[32] Huguenard JR, Prince DA (1992). A novel T-type current underlies prolonged Ca(2+)-dependent burst firing in GABAergic neurons of rat thalamic reticular nucleus. J. Neurosci..

[33] Pan MK (2016). Neuronal firing patterns outweigh circuitry oscillations in parkinsonian motor control. J. Clin. Invest..

[34] Ortuno T, Grieve KL, Cao R, Cudeiro J, Rivadulla C (2014). Bursting thalamic responses in awake monkey contribute to visual detection and are modulated by corticofugal feedback. Front. Behav. Neurosci..

[35] Mc Alonan K, Brown VJ (2016). The thalamic reticular nucleus: more than a sensory nucleus?. Neuroscientist.

[36] Llinas RR, Steriade M (2006). Bursting of thalamic neurons and states of vigilance. J. Neurophysiol..

[37] Li Y (2020). Distinct subnetworks of the thalamic reticular nucleus. Nature.

[38] Steriade M (2003). The corticothalamic system in sleep. Front. Biosci..

[39] Zaman T (2011). Cav2.3 channels are critical for oscillatory burst discharges in the reticular thalamus and absence epilepsy. Neuron.

[40] McAlonan K, Cavanaugh J, Wurtz RH (2006). Attentional modulation of thalamic reticular neurons. J. Neurosci..

[41] Guido W, Weyand T (1995). Burst responses in thalamic relay cells of the awake behaving cat. J. Neurophysiol..

[42] Mukherjee P, Kaplan E (1995). Dynamics of neurons in the cat lateral geniculate nucleus: in vivo electrophysiology and computational modeling. J. Neurophysiol..

[43] Akeju O, Brown EN (2017). Neural oscillations demonstrate that general anesthesia and sedative states are neurophysiologically distinct from sleep. Curr. Opin. Neurobiol..

[44] Crunelli V, Leresche N (1991). A role for GABAB receptors in excitation and inhibition of thalamocortical cells. Trends Neurosci..

[45] Ulrich D, Huguenard JR (1996). Gamma-aminobutyric acid type B receptor-dependent burst-firing in thalamic neurons: a dynamic clamp study. Proc. Natl Acad. Sci. USA.

[46] Crick F (1984). Function of the thalamic reticular complex: the searchlight hypothesis. Proc. Natl Acad. Sci. USA.

[47] Halassa MM (2014). State-dependent architecture of thalamic reticular subnetworks. Cell.

[48] Nakajima M, Schmitt LI, Halassa MM (2019). Prefrontal cortex regulates sensory filtering through a basal ganglia-to-thalamus pathway. Neuron.

[49] De la Casa LG, Mena A, Ruiz-Salas JC (2016). Effect of stress and attention on startle response and prepulse inhibition. Physiol. Behav..

