# Supplementary material for: Involvement of the thalamic reticular nucleus in prepulse inhibition of acoustic startle
Source: Transl Psychiatry. 2021 Apr 24;11:241. doi: 10.1038/s41398-021-01363-1 (PMC8068728; doi:10.1038/s41398-021-01363-1)
Supplement: Supplementary file 1 — Supplementary materials [file 41398_2021_1363_MOESM1_ESM.doc]

**Supplementary materials:**

**Surgery**

For fiber photometry, 200nL of AAV_2/9_-DIO-GCaMP6f was injected into the audTRN subregion in PV-Cre mice. After injection of AAV_2/9_-DIO-GCaMP6f, an optical fiber (outer diameter (OD) of 200μm, numerical aperture (NA) of 0.37, Inper) was placed 100 μm above the injection site.

For chemogenetic manipulations, 200nL of AAV_2/9_-DIO-hM4Di-mCherry orAAV_2/9_-DIO-mCherry was injected into the audTRN subregion in PV-Cre mice. Three weeks after surgery, mice were intraperitoneally injected with clozapine-N-oxide (CNO)(3mg per kg, Sigma Aldrich) half-hour before behavioral test.

For retro-tracing of MG, 200nL of retro beads (Lumafluor) were injected into MG of C57BL/6J mice 3weeks before immunostaining.

For cannula infusion experiment, cannulas were implanted into audTRN or MG in C57BL/6J mice. Two weeks after surgery, 300nL of NiCl2 (6mM, Sigma Aldrich) or CGP55845 (0.1mM, Sigma Aldrich) were infused into audTRN or MG with micro medicine infusion pump 30 min before behavioral test (Vehicle: artificial cerebrospinal fluid(ACSF)).

**Slice preparation**

As previously described^1^, male mice (aged 8- to 12-week) were anesthetized with sodium pentobarbital and then decapitated. The brains were removed quickly and placed into ice-cold modified ACSF containing 250mM sucrose, 26mM NaHCO3, 10mM glucose, 10mM MgSO4, 2mMKCl, 1.3mM NaH2PO4, and 0.2mM CaCl2. Slices containing the TRN or MG (300 µm) were prepared in ice-cold modified ACSF using a VT-1200S vibratome (Leica, Germany), transferred to the storage chamber containing regular ACSF (126mMNaCl, 26mM NaHCO3, 10mM glucose, 3mM KCl, 2mM CaCl2, 1.25mM NaH2PO4, and 1mM MgSO4), and allowed to recover at 34°C for 30 min and then at room temperature (25 ± 1°C) for one hour before recording. During the slice preparation, all solutions were saturated with 95% O2/5% CO2 (vol/vol).

**Fiber photometry**

The fiber photometry system has been described previously^2^. Briefly, a 488-nm laser beam (OBIS 488LS, Coherent) was reflected off a dichroic filter (MD498, Thorlabs), focused by a ×10 objective lens (numerical aperture (NA) of 0.3; Olympus), and then coupled to an optical commutator (Doris Lenses) to record Ca^2+^ signals. The commutator and implanted fiber were connected by a 2-m optical fiber (outer diameter (OD) of 200 µm, NA of 0.37). The laser power at the tip of the optical fiber was adjusted to 0.01mW to decrease bleaching. The GCaMP6f fluorescence was bandpass filtered (MF525-39, Thorlabs). An amplifier was used to convert the photomultiplier tube current output to a voltage signal, which was further digitized at 100Hz and recorded using a Power 1401 digitizer and the software Spike2 (CED).

**Immunostaining**

As previously described^3^, after deep anesthesia, mice were perfused with ice-cold saline followed by 4% paraformaldehyde (PFA) in 0.1 M PBS, pH 7.4. Brains were removed, post-fixed overnight in 4% PFA at 4°C, and transferred to 30% sucrose in 0.1M PBS, pH 7.4. Coronal sections (30 µm) containing the audTRN were cut on a cryostat (Leica CM3050 S). After washing three times in 1% TritonX-100 in PBS (PBST) or Tris-buffered saline (TBS)-Tween 20, the sections were incubated in blocking buffer containing 3% bovine serum albumin and 5% normal goat serum in PBST or TBS-Tween 20 for 1 h at room temperature, and then with primary antibodies in blocking buffer overnight at 4°C (rabbit anti-c-Fos (Millipore ABE457; 1:400) and mouse anti-PV (Swant PV 235; 1:2,000)). After washing three times with PBST or TBS-Tween 20, the sections were incubated with Alexa Flour 594-conjugated anti-mouse IgG (Thermo Fisher A21203; 1:1,000) or Alexa Flour 488-conjugated anti-rabbit IgG (Thermo Fisher A11034; 1:1,000) secondary antibodies at room temperature for 1 h. After another three wash cycles in PBST or TBS-Tween 20, sections were mounted with Pro-Long anti-fade medium (Thermo Fisher). Fluorescent images were collected using a confocal microscope (Nikon A1).

Besides, for c-Fos staining, C57BL/6J mice were sacrificed 90 min after the PPI paradigm.

**Reference:**

1. Luo ZY *et al.* Erbin in Amygdala Parvalbumin-Positive Neurons Modulates Anxiety-like Behaviors. *Biol Psychiatry* 2019.

2. Dong P *et al.* A novel cortico-intrathalamic circuit for flight behavior. *Nat Neurosci* 2019; **22**(6)**:** 941-949.

3. Hu NY *et al.* Expression Patterns of Inducible Cre Recombinase Driven by Differential Astrocyte-Specific Promoters in Transgenic Mouse Lines. *Neurosci Bull* 2020; **36**(5)**:** 530-544.

**Supplementary figures and figure legends:**

**
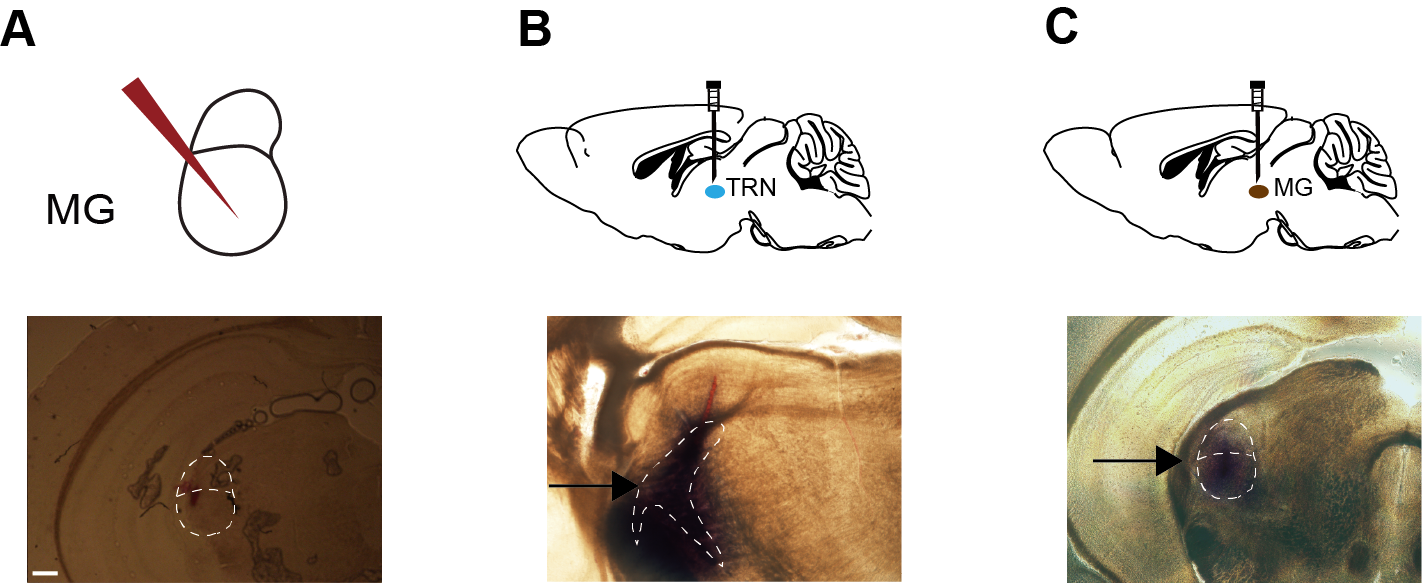
**

**Supplementary Figure S1. Examples of injection sites**

(A) Injection of the retro tracing experiment. Related to Figure 1. RBs were injected into MG of C57 mice. The border of MG was marked by dotted lines. Scale bar: 200 μm. (B) Representative infusion site(as indicated by the arrow) in the mouse audTRN. Related to Figure 4. The border of TRN was marked by dotted lines. The trypan blue staining showed the spread of drugs was limited only to the audTRN. (C) Representative infusion site (as indicated by the arrow) in the mouse MG. Related to Figure 5. The border of MG was marked by dotted lines. The trypan blue staining showed the spread of drugs was limited only to the MG.


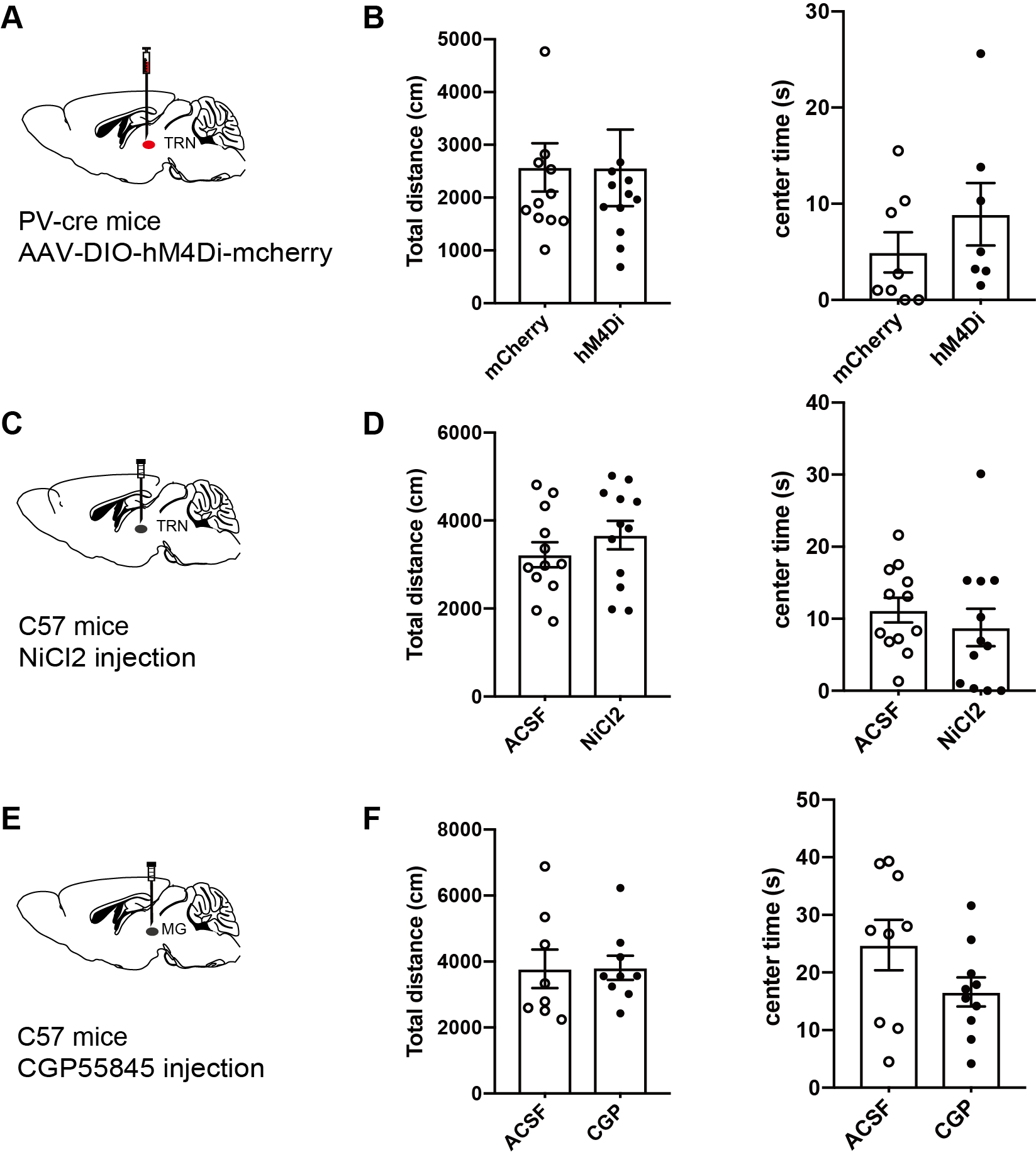


**Supplementary Figure S2. No effects of inhibiting PV+ neurons, NiCl2 and GABAB receptor antagonist on locomotion**

(A) Strategy of chemogenetic Inhibition of PV+ neurons in audTRN. A Cre-dependent AAV for expression of hM4Di was bilaterally injected into the audTRN in PV-cre mice. Related to Figure 3. (B) Application of CNO (3 mg/kg) to mice that expressed hMD4i in audTRN PV neurons had no effect on total distance in 30min OFT (student’s t-test, t_22_=0.0097, P=0.9923, N= 12 per group) or center time in 5min OFT (student’s t-test, t_13_=1.0560, P=0.3104, N_mCherry_=8 mice, N_hM4Di_=7 mice). (C) Schematic of NiCl2-treated experiment. Cannulas were unilaterally implanted into audTRN of C57 mice. After recovery, mice were given 6mM NiCl2 or ACSF 30min before OFT. Related to Figure 4. (D) Infusion of 6mM NiCl2 to audTRN of mice had no effect on total distance in 30min OFT (student’s t-test, t_22_=1.0370, P=0.3109, N= 12 per group) or center time in 5min OFT (student’s t-test, t_22_=0.7717, P=0.4485, N=12 mice per group). (E) Schematic of CGP55845-treated experiment. Cannulas were unilaterally implanted into audTRN of C57 mice. After recovery, mice were given 0.1mM CGP55845 or ACSF 30min prior to OFT. Related to Figure 5. (D) Infusion of 0.1mM CGP55845 to audTRN of mice had no effect on total distance in 30min OFT (student’s t-test, t_15_=0.0461, P=0.9638, N_Ctrl_=8 mice, N_CGP_=9 mice) or center time in 5min OFT(student’s t-test, t_17_=1.6120, P=0.1148, N_Ctrl_=9 mice, N_CGP_=10 mice).
